# Online cognitive behaviour therapy for asthma-related anxiety: a randomised controlled trial

**DOI:** 10.1136/thorax-2025-223886

**Published:** 2026-01-06

**Authors:** Marianne Bonnert, Stephen Nash, Erik M Andersson, Sten Erik Erik Bergström, Jenny Görling, Christer Janson, Josefin Särnholm, Catarina Almqvist

**Affiliations:** 1 Centre for Psychiatry Research, Department of Clinical Neuroscience, Karolinska Institutet, Stockholm, Stockholm County, Sweden; 2 Department of Medical Epidemiology and Biostatistics, Karolinska Institutet, Stockholm, Stockholm County, Sweden; 3 Stockholm Health Care Services, Region Stockholm, Stockholm, Stockholm County, Sweden; 4 Division of Psychology, Department of Clinical Neuroscience, Karolinska Institutet, Stockholm, Stockholm County, Sweden; 5 Pediatric Allergy and Pulmonology Unit at Astrid Lindgren Children’s Hospital, Karolinska University Hospital, Stockholm, Stockholm County, Sweden; 6 Department of Medical Sciences: Respiratory Medicine, Uppsala University, Uppsala, Sweden; 7 Center for Behavioral Cardiovascular Health, New York-Presbyterian/Columbia University Irving Medical Center, New York, New York, USA

**Keywords:** Asthma, Complementary Medicine, Psychology

## Abstract

**Objective:**

Anxiety affects up to one-third of adults with asthma and is linked to poorer disease outcomes and reduced quality of life. This study evaluated the efficacy of therapist-guided, internet-delivered cognitive behavioural therapy (ICBT) versus treatment as usual plus medical education for reducing asthma-related anxiety.

**Methods:**

A randomised controlled trial was conducted including 90 adult participants with anxiety related to asthma. ICBT was therapist-guided and lasted 8 weeks. The primary outcome, the Catastrophising about Asthma Scale, was assessed from pretreatment to the primary endpoint at 16 weeks. Secondary outcomes included asthma control, avoidance behaviour and quality of life. Forced expiratory volume in 1 s (FEV_1_) was collected using a digital spirometer.

**Results:**

ICBT demonstrated a significantly larger reduction in catastrophising about asthma than the control group (mean difference –18.53, 95% CI –25.54 to –11.53, p<0.001). Asthma control, avoidance behaviour, quality of life and other key outcomes improved significantly more in the ICBT group compared with controls. No changes in FEV_1_ were observed. Improvements were sustained at 6 months follow-up.

**Conclusion:**

ICBT effectively and safely reduces catastrophising about asthma, improves asthma control, avoidance behaviour and quality of life and represents a promising adjunct to routine medical care for patients with asthma complicated by anxiety.

**Trial registration number:**

Clinicaltrials.gov (ID: NCT04230369).

**WHAT IS ALREADY KNOWN ON THIS TOPIC**

- Anxiety affects up to one third of adults with asthma and is linked to poorer asthma control, higher symptom burden and reduced quality of life.
- Prior cognitive behavioural therapy (CBT) studies in asthma have been small, suffered high dropout and focused on comorbid panic disorder rather than anxiety elicited by asthma symptoms (breathlessness, fear of exacerbations, avoidance).
- Asthma-specific anxiety—ranging from sub-clinical worry and hypervigilance to panic—is under-recognised and untreated in routine care.

**WHAT THIS STUDY ADDS**

- This is the first adequately powered randomised controlled trial of a fully remote CBT programme designed specifically to reduce asthma-related anxiety in adults.
- Compared with usual care plus medical education, internet-delivered CBT (ICBT) produced significant and clinically meaningful reductions in asthma-specific anxiety, avoidance behaviours and overall symptom burden, alongside improved asthma control and quality of life.
- All benefits were sustained at 6‐month follow-up, with no negative effects on lung function, confirming both the safety and durability of the intervention.

**HOW THIS STUDY MIGHT AFFECT RESEARCH, PRACTICE OR POLICY**

- Demonstrates that fully remote ICBT is a scalable, accessible adjunct to standard asthma care for targeting modifiable psychological drivers of poor asthma outcomes.
- Supports multidisciplinary care models that integrate digital psychological interventions, especially in resource-limited settings or where specialist mental health access is scarce.
- Provides evidence to inform guidelines and health‐policy decisions on incorporating remote CBT into routine asthma care pathways.

## Introduction

 Individuals with asthma often experience distressing symptoms such as wheezing, dyspnoea, hoarseness and chest tightness, which overlap with symptoms commonly associated with anxiety.^1^ The association between asthma and anxiety is well-established^2 3^ and has been associated with poor asthma control, reduced medical adherence, increased healthcare use and diminished quality of life.^3 4^ Although treatment guidelines emphasise addressing anxiety in asthma, evidence for psychosocial interventions remains limited.^5 6^

### A treatment model for anxiety related to asthma

Individuals with heightened levels of anxiety often struggle to distinguish real asthma triggers from harmless bodily sensations, leading to unnecessary avoidance behaviours and reduced quality of life.^7 8^ This pattern reflects the fear-avoidance cycle commonly seen in anxiety disorders, where avoidance temporarily reduces fear but reinforces anxiety and disability over time. Exposure-based cognitive behavioural therapy (CBT) effectively targets this cycle.^1^ In asthma, where breathlessness is central, anxiety may heighten symptom perception by influencing how the brain processes respiratory signals.^3^ Recent theoretical models suggest that an exaggerated avoidance behaviour in chronic diseases may be driven by maladaptive expectations, shaped by past experiences and that the brain makes prediction errors in processing ambiguous signals, which can amplify symptom perception and make symptoms feel worse than they are.^9^ Targeting maladaptive expectations, such as catastrophising about asthma symptoms, may therefore be key in treating asthma-related anxiety. This is in line with contemporary theories that emphasise expectancy violation as a central mechanism of change in exposure-based CBT for anxiety.^10^

### Internet-delivered CBT for anxiety related to asthma

We have developed an exposure-based CBT for anxiety related to asthma to address the need in the asthma population.^11 12^ To enhance accessibility, we adapted the treatment into an internet-delivered CBT (ICBT).^13^ Previous results demonstrated feasibility, acceptability and safety of ICBT and promising clinical effects, suggesting its potential as an effective intervention.^11 13^

The aim of this randomised controlled trial (RCT) was to evaluate the efficacy of ICBT for individuals experiencing anxiety related to asthma, compared with treatment as usual plus medical education (TAU+ME). We hypothesised that ICBT would be more effective than TAU+ME in improving catastrophising about asthma (primary outcome), asthma control, fear of asthma symptoms, excessive avoidance behaviours, perceived stress, worry, anxiety sensitivity, health concerns, insomnia, mood and quality of life. We also hypothesised that ICBT would improve physical lung function, as an indirect consequence of reduced asthma-related anxiety and enhanced self-management following ICBT.

## Methods

### Study design

The RCT was conducted at Karolinska Institutet in Stockholm, Sweden, using a nationwide recruitment strategy. The study was originally launched in February 2020 but was paused shortly thereafter due to the COVID-19 pandemic. Recruitment was subsequently restarted with a new study sample. Participants were recruited from 3 February 2023 to 2 June 2024. Final 6-month follow-up data were collected on 19 January 2025. A total of 90 participants were randomised 1:1 to ICBT or TAU+ME. All participants were Swedish residents, and all study procedures were conducted online, minimising study staff influence on outcome assessments. This study is reported according to the Consolidated Standards of Reporting Trials checklist for non-pharmacological trials. The study received ethical approval by the Swedish Ethical Review Authority in January 2020 (ID: 2019–05985; 2022-01117-02), is preregistered at Clinicaltrials.gov (ID: NCT04230369), and a study protocol has been published.^14^

### Participants

For eligibility, participants had to meet the following criteria: (a) self-reported asthma diagnosed by a physician and regular use of prescribed asthma medication ≥1 year, (b) age ≥18 years, (c) some anxiety related to asthma, defined as fear or avoidance related to asthma symptoms assessed through screening and verified in clinical interview, (d) no recent change in psychotropic medication, (e) no severe psychiatric disorder, (f) no severe respiratory disease other than asthma, (g) no ongoing psychotherapy, (h) no severe cognitive impairment and (i) no significant difficulties reading or writing Swedish, see online supplemental 1.1–1.2 for details.

Participants were nationally recruited through advertisements and enrolled online by completing screening measures and providing electronic informed consent. Eligible applicants underwent a phone-based assessment by a clinical psychologist to exclude conditions requiring urgent psychiatric care or likely to hinder treatment (eg, psychosis, addiction, severe depression, cognitive impairment, language difficulties). During the interview, the psychologist confirmed participants’ informed consent and understanding. Medical concerns were reviewed with a respiratory physician (CJ) before a final decision was made. Excluded applicants were informed by phone and referred to appropriate care if needed. Eligible participants were randomised after completing all preassessments by an external researcher using a computer-generated sequence via random.org. See online supplemental (procedures 1.1–1.4) for further details.

### Intervention

Both groups were instructed to use their prescribed controller medication and permitted to use any other concurrent treatment as appropriate. For more detail and overview of the interventions, see online supplement procedure 1.5 and table S1.

The treatment consisted of eight weekly online modules with therapist support via text from four CBT-trained licensed psychologists, supervised by the lead author (MB). Therapists provided feedback on homework and answered the participants’ questions. An asthma specialist was available for consultation throughout. Modules included texts, case examples and strategies for exposure to feared asthma-related situations (eg, brisk walking or public speaking despite coughing risk). Regular medication use and avoidance of known triggers (eg, smoke, allergens) were emphasised. Further treatment details are available elsewhere.^13 14^

Participants in the control group continued to receive standard, guideline-based asthma care, including medication management and routine follow-up with nurse-led or physician-led reviews and individualised treatment plans in accordance with national recommendations. In addition, they accessed an online module with ME on asthma and a reminder to follow prescribed medication—identical to information in the first module in the ICBT—to control for any effect of increased medical adherence. The control group did not receive therapist support and was offered ICBT after 16 weeks. See online supplemental 1.5–1.6 for further details.

### Data collection

Participants completed online assessments at screening, baseline (pretreatment), week 8 (post treatment), and week 16 (primary endpoint). Some measures, including the primary outcome, were collected weekly during treatment. The treatment group also completed a 6-month follow-up. Forced expiratory volume in 1 s (FEV₁) was measured twice daily with a digital spirometer for 10 days at baseline, post treatment and 2-month follow-up. *For the schedule, see* online supplemental table S2.

### Outcomes

The primary outcome was the between-group difference in the rate of change (slope) in the Catastrophizing about Asthma Scale (CAS) total scores from baseline to 16 weeks. The CAS is a validated 21-item scale of catastrophising thoughts related to asthma (score range 0–84).^15^ Higher scores indicate greater asthma-related anxiety.

Secondary outcomes: the Asthma Control Test (ACT), a validated 5-item questionnaire evaluating symptom severity.^16^ The Fear of Asthma Symptom Scale (FAS) assesses fear of asthma symptoms, while the Asthma Behavior Checklist (ABC) measures behavioural avoidance due to fear of asthma symptoms.^12^ The Brunnsviken Brief Quality of Life Scale (BBQ) measured general life quality.^17^ Anxiety sensitivity was evaluated using the Anxiety Sensitivity Index (ASI), a 16-item scale.^18^ Worry levels were assessed with the 16-item Penn State Worry Questionnaire (PSWQ),^19^ and health concerns were measured with the Short Health Anxiety Inventory (SHAI).^20^ The Insomnia Severity Index (ISI) assessed sleep problems.^21^ Perceived stress was measured using the Perceived Stress Scale (PSS-10).^22^ Depression screening was conducted using the Patient Health Questionnaire (PHQ-9).^23^ Lung function (FEV_1_) was measured using the AsthmaTuner digital device and app for spirometry.^24^ Self-reported data on other treatments and healthcare visits were collected at 8 weeks and 16 weeks. See the Study Protocol for further details on outcome measures.^14^ For process outcomes related to the experience of the ICBT, the Working Alliance Inventory (WAI) was used to measure therapeutic alliance at week 2, and^25^ the Client Satisfaction Questionnaire (CSQ-8) assessed treatment satisfaction at week 8.^26^ Both groups were evaluated using the Subjective Adequate Relief Questionnaire to assess perceived overall symptom change at week 8.^27^ Reports about any adverse events were collected after treatment completion. Information on concurrent treatments and healthcare contacts was collected at 8 weeks and 16 weeks, see online supplement 1.6.

### Statistical analysis and power

We estimated that a sample of 90 participants randomised in 1:1 ratio would provide 80% power (α=0.05) to detect a moderate between group standardised mean difference (Cohen’s d=0.6) on the primary outcome (CAS) at 16 weeks, allowing for 25% attrition. Full details are available in the published study protocol.^14^

All analyses were prespecified in the study protocol before trial completion and before the statistician accessed unblinded data.^14^ The primary outcome (CAS) was analysed using a linear mixed-effects model with all data up to week 16, including a treatment-by-time interaction and a random intercept for each participant. No other covariate adjustment was used. Treatment effect was estimated by the interaction parameter and interpreted as the difference in weekly change in CAS. We used elapsed time (in weeks) from the start of treatment in all regression models. Standardised measures were obtained by dividing all measurements by the pooled SD of the baseline score. All other weekly measurements were analysed in the same way. We also report change from baseline to 16 weeks estimated from a linear regression model, adjusted for baseline score. Furthermore, we conducted a dose-response analysis, using the number of ICBT modules completed as continuous exposure variable. Participants randomised to TAU+ME were set to zero. The same linear mixed-effects model was used, interpreting the interaction as the change in slope per additional module completed. All analyses were performed in Stata V.18.0.

## Results

**Figure 1:**
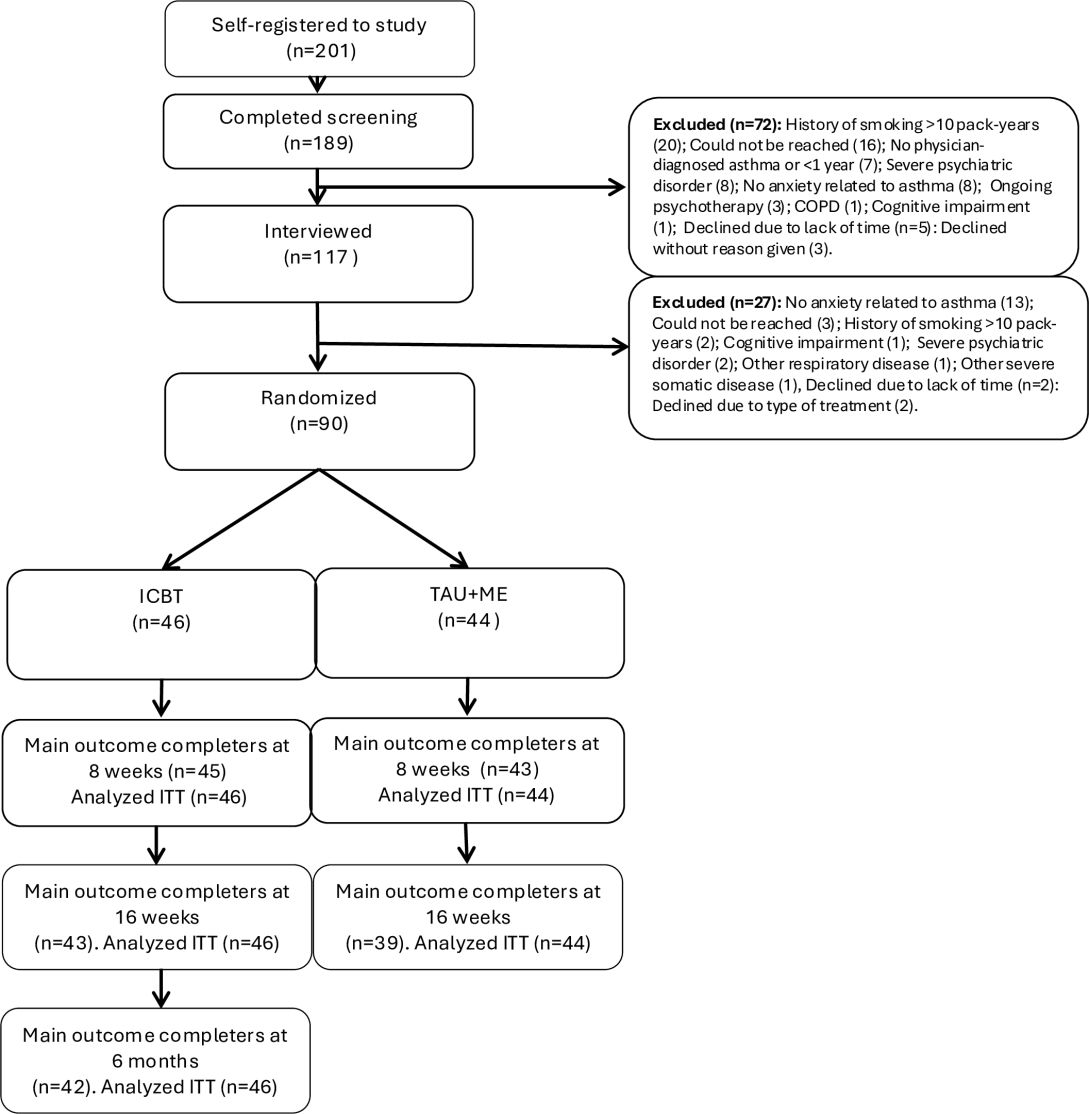
Participant flow through the study. COPD, chronic obstructive pulmonary disease; ICBT, internet-delivered cognitive behavioural therapy; ITT, intention-to-treat; TAU+ME, treatment as usual + medical education.

The participants were randomised in a 1:1 ratio to ICBT (n=46) or TAU+ME (n=44). See figure 1 for study flow. The groups were well balanced at baseline, across demographic, clinical and treatment-related characteristics. See table 1 and online supplemental tables S3, S4. Sex was determined using the Swedish personal identity number, which encodes biologic sex at birth as registered in the national population registry. Participants were predominantly women (85%), with a mean age of 48 years (SD=15). Most were employed, university educated and married or cohabiting. The majority had long-standing asthma (>10 years). Asthma severity was well balanced between groups, with only about one in six showing well-controlled asthma (ACT≥20). Approximately half met criteria for an anxiety disorder and one quarter for a mood disorder. No statistically significant differences were observed between groups at baseline.

**Table 1:** Baseline characteristics of participants

	ICBT (n=46) n (%)	TAU+ME (n=44) n (%)
Women	40 (87)	36 (82)
Age, years, mean (SD), min-max	49.1 (15.3), 21–77	46.8 (15.8), 23–77
Occupational status		
Working full time	25 (54)	22 (50)
Working part time	9 (20)	9 (20)
Student	2 (4)	6 (14)
Retired	7 (15)	6 (14)
Other	3 (7)	1 (2)
Marital and family status		
Married or de facto	34 (74)	27 (61)
Parent	30 (65)	29 (66)
Educational level		
University	32 (70%)	31 (70%)
Upper secondary school	10 (22%)	8 (18%)
Other	4 (9%)	4 (9%)
Asthma severity		
Poorly controlled (ACT <15)	18 (39%)	15 (34%)
Partially controlled (ACT 15-19)	20 (44%)	22 (50%)
Controlled (ACT ≥20)	8 (17%)	7 (16%)
Duration of asthma		
>10 years	28 (61%)	25 (57%)
2–10 years	15 (33%)	16 (36%)
<2 years	3 (6%)	3 (7%)
Asthma medication		
SABA	23 (50%)	22 (50%)
LABA	4 (9%)	1 (2%)
ICS	16 (35%)	13 (30%)
ICS+LABA	31 (67%)	26 (59%)
SAMA	2 (4%)	0
LAMA	5 (11%)	0
LAMA+LABA+ICS	1 (2%)	0
LTRA	20 (44%)	12 (27%)
Biologics	3 (7%)	1 (2%)
Allergy		
Allergic rhinitis	26 (57)	31 (70)
Animal dander	19 (41)	25 (57)
Food	22 (48)	18 (41)
Other*	11 (25)	11 (24)
No allergies	8 (17)	4 (9)
Medical history		
Atopic dermatitis	10 (22)	5 (11)
Irritable bowel syndrome	4 (8)	7 (15)
Migraine	4 (8)	5 (11)
Hypertension	3 (6)	2 (4)
Endometriosis	1 (2)	2 (4)
Diabetes	2 (4)	0
Fibromyalgia	1 (2)	1 (2)
Other†	21 (43)	14 (30)
>Two diagnoses	5 (10)	5 (11)
Without additional diagnosis	22 (45)	22 (47)
Smoking		
History of smoking‡	20 (43.5)	19 (43.2)
Never smoker	26 (56.5)	25 (56.8)
Psychiatric disorders§		
Mood disorders (F30–39)	11 (23.9)	7 (15.9)
Anxiety disorders (F4–48)	23 (50)	13 (29.5)
Self-reported worry, insomnia or stress	32 (70)	25 (57)
Psychotropic medicine¶	12 (26)	10 (23)

No statistically significant differences were observed between the ICBT and TAU+ME groups across any baseline characteristics.

* Mould, dust mites, antibiotics, nickel allergy, wasps.

† Epilepsy, trigeminal neuralgia, chronic pain, Ehlers-Danlos syndrome, lipoedema, vestibulodynia, psoriasis, osteoarthritis, autoimmune hepatitis, hypothyroidism, atrial fibrillation, atrophic gastritis, microscopic colitis, rosacea, fall injury, ichthyosis.

‡ Includes previous and occasional smokers.

§ Clinical assessment using Mini International Neuropsychiatric Interview.

¶ Selective serotonin reuptake inhibitors (sertraline, citalopram, fluoxetine), other antidepressants (mirtazapine, duloxetine, buspirone), mood stabilisers (lithium) and stimulants (methylphenidate).

ACT, Asthma Control Test; ICBT, internet-delivered cognitive behavioural therapy; ICS, inhaled corticosteroids; LABA, long-acting beta2-agonists; LAMA, long-acting muscarinic antagonists; LTRA, leukotriene receptor antagonist; SABA, short-acting beta2-agonists; SAMA, short-acting muscarinic-antagonist; TAU+ME, treatment as usual + medical education.

At post treatment, outcomes were assessed for all randomised participants according to the intention-to-treat principle. At week eight, data attrition was 2% in each group (ICBT: n=1; TAU+ME: n=1). At week 16, attrition was 6.5% (n=3) in ICBT and 11% (n=5) in the control group. The ICBT group completed 308 out of 322 weekly assessments (95.7%), while the control group completed 278 out of 308 (90.3%). At the 6-month follow-up, attrition was 7% (n=3) in ICBT. Due to the low attrition, missing data were not imputed.

On average, participants completed 80% of the treatment content over 8 weeks, with 40 participants (86%) considered treatment completers. See online supplemental table S6 for details. The mean therapist time per week was 9.2 min (SD=5.5).

### Primary outcome

**Figure 2:**
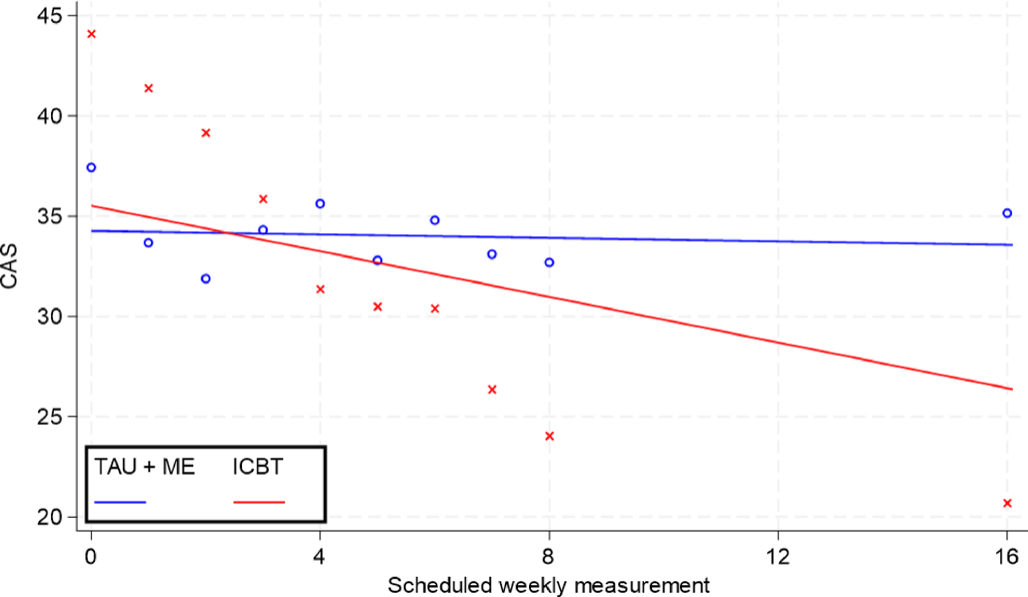
Weekly change in the primary outcome Catastrophising about Asthma Scale (CAS) across treatment groups from baseline to primary endpoint. ICBT, internet-delivered cognitive behavioural therapy; TAU+ME, treatment as usual plus medical education.

The mean weekly change in the primary outcome (CAS) was –0.57 in ICBT and −0.04 in the control group, yielding a treatment effect of –0.53 /week (95% CI –0.74 to–0.31; p<0.001). See table 2 for detailed results. At 16 weeks, the ICBT group showed greater improvement on CAS than the control group (mean difference –18.53; 95% CI –25.54 to –11.53; p<0.001). The standardised mean difference (Cohen’s d 1.17, 95% CI 0.73 to 1.61) represents a large effect in favour of ICBT (figure 2). Mean CAS in the ICBT group at 6-month follow-up (22.9, SD=19.0) indicated stability from post-treatment (24.0, SD=16.7) and primary endpoint (20.7, SD=16.6). For complete results, including detailed tables, see the online supplement (section 2) Results.

**Table 2:** Patient-rated outcomes from baseline to the primary endpoint at 16 weeks

Outcome	Baseline mean(SD)		16 weeks mean(SD)		Treatment effect (95% CI)	P value
	ICBT	TAU+ME	ICBT	TAU+ME		
CAS	44.11 (15.78)	37.43 (15.29)	20.70 (16.58)	35.15 (21.28)	−18.53 (−25.54 to −11.53)	<0.001
ACT*	15.13 (4.32)	16.39 (3.98)	19.95 (4.24)	17.56 (4.42)	2.80 (1.00, 4.59)	0.003
FAS	29.46 (12.07)	23.93 (11.82)	12.53 (11.99)	20.62 (12.57)	−11.03 (−15.58 to −6.47)	<0.001
ABC	27.33 (11.97)	26.82 (11.15)	17.51 (8.77)	25.79 (12.71)	−8.50 (−12.37 to −4.64)	<0.001
BBQ*	54.43 (19.99)	55.41 (19.80)	66.65 (19.62)	56.54 (19.85)	11.36 (4.13 to 18.59)	0.002
ASI	22.39 (10.81)	22.61 (11.72)	13.26 (9.18)	19.23 (11.15)	−6.27 (−9.08 to to −3.46)	<0.001
PSWQ	52.91 (13.13)	52.89 (12.58)	46.71 (11.75)	52.45 (13.10)	−5.63 (−9.02 to −2.24)	0.001
SHAI	21.52 (8.48)	20.14 (9.83)	15.70 (8.08)	18.44 (10.02)	−4.38 (−6.56 to −2.19)	<0.001
ISI	11.37 (6.76)	10.61 (6.24)	8.76 (6.35)	9.92 (5.63)	−2.12 (−4.08 to −0.15)	0.035
PSS-10	18.20 (7.11)	18.14 (7.13)	13.43 (8.36)	15.87 (7.38)	−2.38 (−5.22 to 0.45)	0.099
PHQ-9	6.72 (4.64)	6.93 (4.78)	4.93 (5.35)	6.68 (5.39)	−1.74 (−4.01 to 0.53)	0.131

* Increase in ACT and BBQ shows improvement. The reported mean difference is the estimated parameter for the randomisation arm in a linear regression model, adjusted for baseline score. A positive number indicates a larger positive change in the ICBT arm (the aim when an increase in the scale represents an improvement in symptoms). A negative number indicates a larger negative change in the ICBT arm (the aim when a decrease in the scale represents an improvement in symptoms). Participants included in estimates: For baseline means, N_ICBT_=46 and N_TAU+ME_ = 44. For 16 weeks means treatment effect estimates, N_ICBT_=43 and N_TAU+ME_ = 39 (except for PSWQ, ISI, PSS10, PHQ9, where N_ICBT_=42 and N_TAU+ME_ = 38).

ABC, Asthma Behavior Checklist; ACT, Asthma Control Test; ASI, Anxiety Sensitivity Index; BBQ, Brunnsviken Brief Quality of Life; CAS, Catastrophizing about Asthma Scale; FAS, Fear of Asthma Symptoms Scale; ICBT, Internet-delivered cognitive behavioral therapy; ISI, Insomnia Severity Index; PHQ-9, Patient Health Questionnaire; PSS-10, Perceived Stress Scale; PSWQ, Penn State Worry Questionnaire; SHAI, Short Health Anxiety Inventory; TAU+ME, Treament as usual+medical education.

### Secondary outcomes

#### Patient reported ratings from baseline to primary endpoint

**Figure 3:**
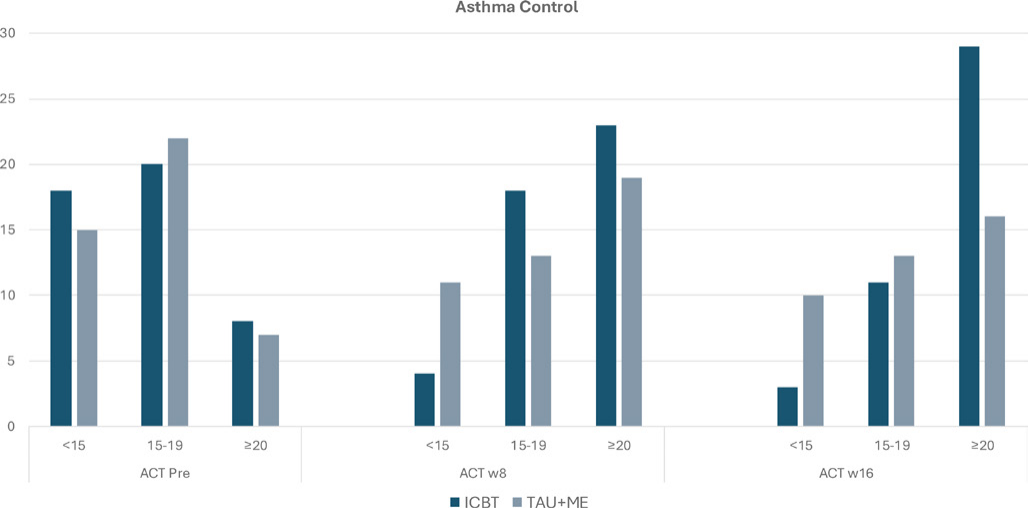
Asthma control (Asthma Control Test (ACT)) scores at baseline (Pre), post treatment (week 8) and the primary endpoint (week 16). ACT <15 = poor control; ACT 15–19 = partially controlled; ACT ≥20 = controlled.^16^ ICBT, internet-delivered cognitive behavioural therapy; TAU+ME, treatment as usual plus medical education.

ICBT improved asthma control (ACT), with a 0.21 greater weekly change than the control group (95% CI 0.12 to 0.30; p<0.001. At 16 weeks, 63.0% of ICBT participants and 36.4% of TAU+ME participants reported controlled asthma (ACT ≥20) (figure 3).

We observed evidence of a beneficial effect of ICBT on nearly all secondary outcomes; fear of asthma symptoms (FAS), avoidance behaviours (ABC), quality of life (BBQ), anxiety sensitivity (ASI), worry (PSWQ), health concerns (SHAI) and sleep (ISI). There was no difference in change between groups for perceived stress (PSS-10) or depression (PHQ-9). See table 2 for detailed results and Supplement (online supplemental table S7) for effect sizes. All improvements were stable at 6 months follow-up in the ICBT group, see online supplemental table S5.

#### Objective measure of lung function from pretreatment to primary endpoint

There was no significant difference between the groups for change in FEV_1_ over time (difference in slopes −0.002; 95% CI −0.009 to 0.005; p=0.58).

### Dose-response

A significant association was observed between the number of modules completed and CAS scores, with each additional module completed associated with a decrease in CAS score (−0.18; 95% CI –0.22 to –0.15, p<0.001), suggesting a dose-response effect of the ICBT. See online supplemental tables S8,S9.

### Treatment experience and subjective adequate relief

ICBT participants reported a strong working alliance with the therapist (WAI mean=30.2/42), indicating high levels of trust, unity, and goal agreement. Overall treatment satisfaction was high (CSQ mean=19.7/24), including those who did not complete the full ICBT. Most ICBT participants (n=40; 87%) reported adequate symptom improvement, compared with 17 (39%) in TAU+ME. See online supplemental 2.4 and online supplemental figure S1. No serious adverse events were reported. See online supplemental 2.5.

### Other treatment

From baseline to 16 weeks, two participants in each group received physiotherapy; additionally, two in the ICBT group saw a psychologist. Urgent asthma-related visits were reported by three ICBT participants (total four visits) and three control participants (total six visits). Most participants reported stable asthma medication use at 8 weeks (ICBT 62%, TAU+ME 66%) and 16 weeks (ICBT 81%, TAU+ME 73%). See online supplemental (2.6) and table S10.

## Discussion

We have conducted an RCT that investigated the efficacy of ICBT for adults with anxiety related to their asthma. We observed significant improvement in catastrophising about asthma, asthma control, fear of asthma symptoms, avoidance behaviours, quality of life, anxiety sensitivity, worry, health concerns and sleep in ICBT compared with TAU + ME. Perceived stress and depression decreased slightly, with no difference between groups. The improvements were stable 6 months after treatment completion. Lung function (FEV_1_) was stable throughout the study period. Data attrition was low, and treatment adherence was high. Participants found ICBT satisfying and leading to adequate relief. Participants in both groups reported receiving other treatment during the study period. However, most participants did not receive any other treatment, indicating the low availability of treatments for this population. The reported adverse events were mostly mild and transient, arising from the challenges ICBT can pose for some patients.

The combined results suggest that ICBT may enhance asthma management by reshaping symptom perception and reducing fear-driven behaviours. Asthma-specific exposure exercises and cognitive restructuring likely helped participants develop more realistic expectations about their condition, reducing catastrophic thinking and promoting more consistent asthma medication use and improved asthma control. In contrast to our hypothesis, we could not observe any changes in FEV_1_. This might have been expected given the brief intervention, as well as previous results in smaller studies.^13 28^ Importantly, there was no deterioration in FEV1, suggesting that while ICBT may not improve lung function, it can enhance symptom perception and is safe, even with exposure to asthma-related symptoms.

Our findings are consistent with the few existing studies demonstrating promising effects of CBT on asthma-related anxiety.^6^ However, unlike these studies, our research included a larger sample size and had low attrition rates, enhancing the reliability of the findings. While previous studies observed improvements in certain key variables,^6^ the current study demonstrated robust and widespread effects across all measured dimensions of anxiety, asthma control and quality of life.

The ICBT approach is novel in the field of asthma. While ICBT has shown promising results in treating various somatic disorders,^29^ it has also proven feasible, safe and effective as an adjunct to medical treatments in conditions such as atrial fibrillation.^30^ Thus, this study provides additional support for the use of ICBT in a multidisciplinary strategy for managing chronic diseases. ICBT complements medical treatment by addressing asthma-specific anxiety that can impair asthma control.

A key advantage of ICBT is its accessibility for patients facing barriers to in-person therapy, such as geographic distance, time constraints or limited access to disease-specific CBT expertise.^29^ By integrating ICBT with standard medical treatment, healthcare providers can offer a flexible and scalable solution that can reach a larger number of patients, ensuring that more individuals benefit from both the psychological and physical aspects of asthma care.

### Strengths and limitations

High adherence suggests that participants found the internet-delivered treatment engaging and feasible, while low attrition reduces the risk of bias from missing data, thereby strengthening the study’s internal validity. A significant dose-response relationship was observed, indicating that greater engagement with the ICBT modules was associated with stronger clinical improvement. However, the limited variation in module completion within the study sample may have resulted in a conservative estimate of this effect. In routine clinical care, where engagement levels are likely to vary more widely, a stronger dose-response relationship might be expected. Additionally, the inclusion of the digital spirometer AsthmaTuner provides an objective measure of lung function, allowing for a more comprehensive evaluation of ICBT’s impact on asthma. Another key strength is that the control group received the same ME as the ICBT group, ensuring that observed differences between groups can be attributed to the psychological intervention rather than disparities in medical information. The study also benefited from being grounded in prior development and feasibility work, which guided the refinement of both the intervention and trial procedures.

However, there are limitations to consider. The study sample mainly comprised self-referred volunteers, predominantly women with relatively high educational attainment and digital literacy. While such characteristics may reflect greater motivation than typically observed in routine asthma care, women are also more likely than men to have asthma and to experience anxiety symptoms,^2 3^ suggesting that this sample reflects the population most affected by asthma-related anxiety. Meta-analytic evidence shows that ICBT for psychiatric disorders produces similar outcomes regardless of referral route, supporting the relevance of self-referred and digitally engaged individuals as the primary users of such interventions.^29^ Another limitation is the inability to independently verify participants’ asthma diagnoses. However, all participants reported having been diagnosed by a named physician at a specific clinic, and their medication lists were consistent with asthma treatment. Moreover, previous research has demonstrated good agreement between self-reported physician-diagnosed asthma and medical records, supporting the validity of this approach.^31^ Participants were not required to meet diagnostic criteria for a specific anxiety disorder. This inclusive approach reflects clinical reality, where subclinical anxiety is common and can meaningfully impair asthma control.^32^ While this may limit diagnostic specificity, it enhances generalisability and mirrors the diverse presentations seen in routine care, thereby increasing the clinical relevance of our findings. Additionally, most outcomes rely on self-report questionnaires, which makes it difficult to verify if the reported outcomes truly reflect changes in the condition. However, many aspects of mental health, quality of life and well-being are inherently subjective, making self-reports one of the best ways to capture these experiences. The TAU+ME control did not account for attention and blinding was not possible, but it reflects standard clinical care, enhancing ecological validity. It is worth noting that in routine clinical care, patients may not consistently receive the same extent or quality of ME as participants in the usual care group, suggesting that the treatment effects observed in this trial may be conservative.

## Conclusions

To our knowledge, this is the first RCT to evaluate the efficacy of ICBT for adults with anxiety related to asthma. We have demonstrated that 8 weeks of ICBT can effectively and safely reduce catastrophising about asthma as well as improve asthma control, fear of asthma symptoms, avoidance behaviours, quality of life, anxiety sensitivity, worry, health concerns and sleep in adults with asthma complicated by anxiety. We conclude that ICBT using exposure exercises to relieve anxiety in asthma can be an effective adjunct to standard medical therapies and asthma management. Further research in routine clinical care could help confirm the generalisability and effectiveness of these findings under real-world conditions.

## Supplementary material

10.1136/thorax-2025-223886 online supplemental file 1

10.1136/thorax-2025-223886 online supplemental file 2

## Data Availability

Data are available upon reasonable request.
