# Supplementary material for: Online cognitive behaviour therapy for asthma-related anxiety: a randomised controlled trial
Source: Thorax. 2026 Jan 6;81(8):e223886. doi: 10.1136/thorax-2025-223886 (PMC13479204; doi:10.1136/thorax-2025-223886)
Supplement: Supplementary file 1 [file thorax-81-8-s001.pdf]

Online cognitive behaviour therapy for asthma-related anxiety: a randomised controlled trial

Bonnert. M. et al. *Thorax* 2025. Doi: 10.1136/thorax-2025-223886

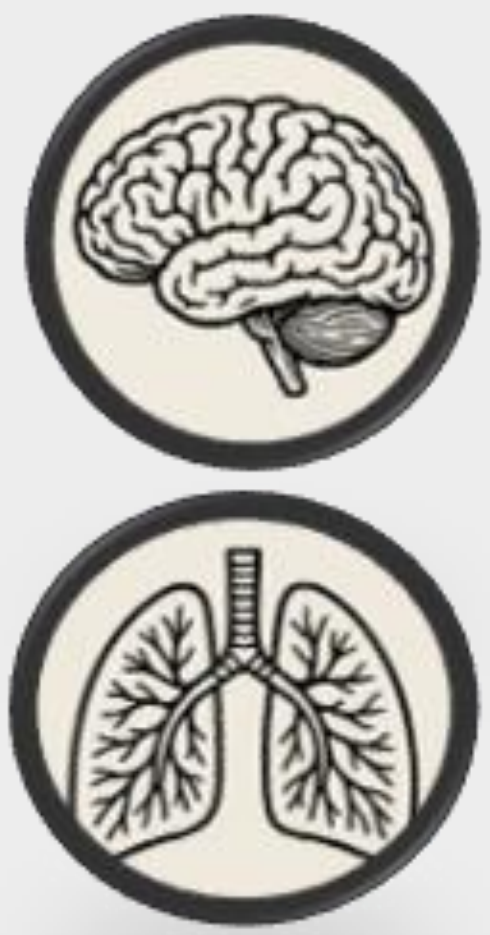

Anxiety

+

Asthma

BACKGROUND

One in three adults with asthma experience anxiety.

Associated with poor asthma control and low quality of life.

METHODS

RCT (N=90): Internet-based cognitive behaviour therapy (ICBT) vs treatment as usual (TAU)

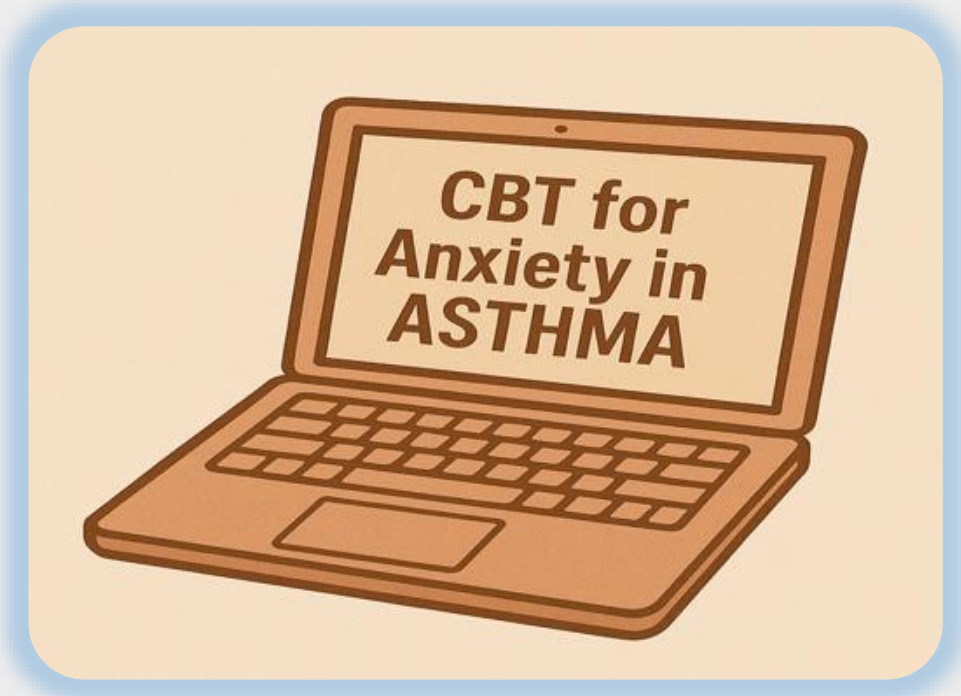

8 weeks ICBT →

RESULTS

Catastrophizing about asthma
↓ Large reduction

Asthma control
↑ Improved

Avoidance behaviours
↓ Reduced

Quality of life
↑ Increased

FEV_1
↔ No change

ICBT improves psychological and functional outcomes without affecting lung function

Thorax

thorax.bmj.com

@ThoraxBMJ

British Thoracic Society BMJ Group
