# Supplementary material for: Online cognitive behaviour therapy for asthma-related anxiety: a randomised controlled trial
Source: Thorax. 2026 Jan 6;81(8):e223886. doi: 10.1136/thorax-2025-223886 (PMC13479204; doi:10.1136/thorax-2025-223886)
Supplement: Supplementary file 2 [file thorax-81-8-s002.pdf]

Supplement to

Online cognitive behaviour therapy for asthma-related anxiety: a randomised controlled trial

1. PROCEDURES

1.1 Online screening

During the online screening, participants completed the Patient Health Questionnaire (PHQ-9) to assess the severity of depressive symptoms. The Alcohol Use Disorders Identification Test (AUDIT) and the Drug Use Disorders Identification Test (DUDIT) were used to identify any substance use problems. Participants also completed a demographic questionnaire. In addition, they answered questions about:

a) the year and clinic of their asthma diagnosis and their current asthma medication.

b) fear or avoidance related to asthma (yes/no).

c) current and past somatic and psychiatric diagnoses, to be further explored during the clinical interview in order to assess whether the severity or nature of these conditions might interfere with participation.

d) smoking history, where ≥10 pack-years of smoking served as an exclusion criterion to avoid the unintentional inclusion of individuals with COPD.

1.2 Clinical interview

The interview was conducted over the phone by a clinical psychologist using the Mini-International Neuropsychiatric Interview (M.I.N.I.) a structured diagnostic interview designed to assess psychiatric disorders according to ICD and DSM criteria.[1] Participants were considered to have asthma-related anxiety if the psychologist judged that fear of asthma symptoms led to avoidance or distress interfering with daily activities. If any uncertainties arose regarding the inclusion criteria in the online screening, these were further clarified during the clinical interview. When needed, medical expertise was sought through consultation with an asthma specialist (co-author CJ).

1.3. AsthmaTuner

Lung function was measured using the AsthmaTuner system (Medituner AB, Stockholm, Sweden), a CE-marked digital tool for asthma self-management that includes a Bluetooth spirometer for FEV_1 assessment. AsthmaTuner has demonstrated feasibility, clinical benefit, and diagnostic accuracy in clinical studies.[2,3] The device connects to a mobile application developed by MediTuner which securely store patient data.

The device was sent to eligible participants after the clinical interview with a manual to start the device and connect to the app, and with the following specific instructions for the current study:

"You will use AsthmaTuner on three separate occasions. Each occasion lasts for 10 days, during which you should use AsthmaTuner twice daily. Try to use it at approximately the same time each day. After reading this manual and downloading the app, you should use AsthmaTuner for the first time. You will then use it again after two and four months, following the same schedule—twice daily for 10 days. You will receive reminders for these sessions. Please do not use AsthmaTuner between these periods. After completing the treatment, you may keep your AsthmaTuner device. If you find it difficult to blow, try to blow steadily and for as long as you can. Simply keep the mouthpiece in your mouth until AsthmaTuner registers your value—you do not need to blow as hard as possible the entire time."

1.4. Randomisation

The randomisation was conducted by a researcher (Dorian Kern, KI) with no connection to this research project. Study codes were consecutively sent to the researcher after completed pre-assessments with the following instruction:

"Randomisation is conducted via random.org using the List Randomizer.

1. *Enter the list of participant IDs and click "Randomize." If the total number is odd, add a **DUMMY** participant.*
2. *Take a screenshot of the results, including the timestamp.*
3. *Enter a list of equal length with ICBT/TAU assignments (50%/50%).*
4. *Take a screenshot of the results, including the timestamp.*
5. *Email both screenshots back.*
6. *Save the results in an Excel file."*

The results were saved by both the external researcher and the study team, double-checked, and found to be error-free.

1. 5 Interventions

To control for education about asthma and the information about the importance to adhere to medication as prescribed, participants in both groups had online access to the same medical education about asthma with a digital worksheet including a plan for medication and how to act during exacerbations. This was included in the first module in the ICBT group, and as an only module for the TAU group. Participants allocated to TAU+ME continued to receive standard asthma care through primary or specialist healthcare services in Sweden, in accordance with national guidelines. Usual care typically includes pharmacological management, periodic asthma reviews, and access to nurse- or physician-led asthma education and follow-up as needed. The frequency and intensity of contact may vary based on asthma severity and individual needs.

Table S1. Overview of interventions

Weekly module	***ICBT***	***TAU+ME***
Module 1	*Medical Education about asthma + plan for medication*<br>*Psychoeducation about anxiety in asthma*<br>*Goal setting*<br>*Mapping of own behaviour*	*Medical Education about asthma + plan for medication*
Module 2	*Differentiate between symptoms*<br>*Affective Labelling*<br>*Challenge one behaviour*	
Module 3	*Exposure education*<br>*Mapping difficult situations*<br>*Exposure exercises*	
Module 4	*Use labelling in exposure*<br>*Medication plan follow-up*<br>*Exposure exercises*	
Module 5	*Asthma and sleep*<br>*Sleep hygiene*<br>*Exposure exercises*	
Module 6	*Worry and asthma behaviours*<br>*Exposure exercises*	
Module 7	*Step up exposure*<br>*Exposure exercises*	
Module 8	*Summary of the treatment and plan forward for sustained effects*	

1.5.1 Development and pilot testing

The ICBT was developed through a multi-stage process described in detail elsewhere.[4,5] The program content and therapist procedures were refined based on feedback from participants, and clinicians during earlier feasibility studies. The version used in the present RCT corresponds to the final iteration from the most recent feasibility trial, with only minor wording updates to improve readability.

1.5.2 Adherence monitoring

The treatment platform automatically recorded when each module was released, opened, and when homework (a worksheet included in each module) was submitted. Participants were required to complete the homework before the next module became available. Homework completion was confirmed either through submission of the worksheet within the platform or through participant messages to the therapist indicating that the tasks had been completed.

1.5.3 Therapist training and treatment fidelity

All therapists were licensed clinical psychologists with prior CBT experience. Before the trial, they received specific training from the lead author (MB), who developed the intervention in two preceding feasibility studies. The training covered CBT principles for anxiety related to asthma, including psychoeducation on asthma and medication, maintaining stable anti-inflammatory treatment, and guideline-consistent use of short-acting bronchodilators (e.g., 15 minutes before exertion or after using affective labelling - a brief 2-3 min exercise to reduce negative reactivity - prior to as-needed use, without delaying medication when indicated).

The asthma-related content and medication strategies were developed in collaboration with asthma specialists (CA and SEB) during earlier feasibility work. An asthma specialist (CJ) was available for consultation during the RCT if clinical questions arose. Therapists received supervision on demand from MB throughout the trial. The internet-based format ensured standardized treatment delivery, and therapists primarily supported engagement and individualized exposure planning, securing fidelity across all four therapists

1.6 Monitoring of concurrent treatments and healthcare contacts

At 8 and 16 weeks, participants reported any additional non-medical treatments related to asthma or anxiety (e.g., psychological, physiotherapeutic, or alternative treatments), acute healthcare visits for asthma, changes in asthma medication (including whether in consultation with a physician), and any healthcare contacts during the previous four weeks, including specialists, general practitioners, nurses, psychologists, social workers, physiotherapists, or other professionals.

Table S2. Overview of outcome assessment points

**Outcome (all were assessed at baseline)**	**Weekly assessment during treatment period**	**Post treatment** *(week 8)*	**Primary endpoint at 2 mo FU**	**6 mo FU** *(ICBT)*
**Primary outcome:** ***Catastrophizing about asthma, CAS***	X	X	X	X
**Secondary Outcomes:** ***Asthma control, ACT***	X	X	X	X
Fear of asthma symptoms, FAS	X	X	X	X
Excessive avoidance, ABC	X	X	X	X
Perceived stress, PSS-10	X	X	X	X
Worry, PSWQ	X		X	X
Anxiety Sensitivity, ASI	X		X	X
Health Anxiety, SHAI	X		X	X
Insomnia, ISI	X		X	X
Depression, PHQ-9	X		X	X
Quality of life, BBQ	X		X	X
FEV1, AsthmaTuner©	X		X	

Note. ICBT=internet-delivered cognitive behavior therapy; TAU+ME =treatment as usual plus medical education; CAS=Catastrophizing about Asthma Scale; ACT=Asthma Control Test; FAS=Fear of Asthma Symptoms scale; ABC=Asthma Behavior Checklist; PSS=Perceived Stress Scale; PSWQ=Penny State Worry Questionnaire; ASI=Anxiety Sensitivity Index; SHAI=Short Health Anxiety Inventory; ISI=Insomnia Severity Index; PHQ= Patient Health Questionnaire; BBQ= Brunnsviken Brief Quality of Life Scale; FEV1=Forced expiratory volume per 1 second. AsthmaTuner© is a registered digital spirometer which participants connect to an App and use at home.

2. RESULTS

2. 1 Results from screening and interview

Table S3. Psychiatric diagnosis (clinical interview + MINI)

	ICBT (N=46)	TAU+ME (N=44)
Depression, n (%)	10 (22%)	7 (16%)
Worry, n (%)	17 (37%)	13 (30%)
Social Anxiety, n (%)	6 (13%)	4 (9%)
Panic Disorder, n (%)	9 (20%)	3 (7%)
Agoraphobia, n (%)	6 (13%)	1 (2%)
OCD, n (%)	0	2 (5%)
Insomnia, n (%)	10 (22%)	7 (16%)
Stress, n (%)	5 (11%)	5 (11%)
PTSD, n (%)	2 (4%)	2 (5%)
ADHD, n (%)	3 (7%)	2 (5%)
Bipolar disorder, n (%)	1 (2%)	0
EUPD, n (%)	0	1 (2%)
Health Anxiety, n (%)	0	1 (2%)
Autism, n (%)	0	1 (2%)

OCD=obsessive compulsive disorder; PTSD=post-traumatic stress disorder; ADHD=attention-deficit/hyperactivity disorder; EUPD= emotionally unstable personality disorder

Table S4. Healthcare contacts in the last 4 weeks at screening

Healthcare Provider	ICBT (N=46), N	TAU (N=44), N
Asthma Specialist (MD), n (%)	9 (19.6%)	8 (18.2%)
Mean visits (SD)	1.2 (0.44)	1.2 (1.07)
General Practitioner, n (%)	15 (32.6%)	21 (47.7%)
Mean visits (SD)	1.3 (0.62)	1.2 (0.52)
Other Physician, n (%)	12 (26.1%)	6 (13.6%)
Mean visits (SD)	1.7 (0.39)	1.7 (0.41)
Registered Nurse, n (%)	14 (30.4%)	13 (29.6%)
Mean visits (SD)	1.7 (0.94)	1.4 (0.96)

2.2 Detailed main results on outcomes

Table S5. Summary of self-reported outcome means at each week from baseline to week 26.

**Mean<br>SD<br>N**	**W 0**	**W 1**	**W 2**	**W 3**	**W 4**	**W 5**	**W 6**	**W 7**	**W 8**	**W 16**	**W 26**
**CAS**											
Mean<br>ICBT<br>SD<br>N	44.11<br>15.8<br>46	41.39<br>17.3<br>46	39.18<br>16.4<br>45	35.86<br>16.2<br>44	31.37<br>18.8<br>43	30.50<br>20.3<br>44	30.40<br>18.6<br>42	26.36<br>18.8<br>44	24.04<br>16.7<br>45	20.70<br>16.6<br>43	22.88<br>19.0<br>42
Mean<br>TAU+ME<br>SD<br>N	37.43<br>15.3<br>44	33.67<br>15.6<br>43	31.88<br>16.4<br>41	34.31<br>19.3<br>39	35.62<br>19.5<br>40	32.79<br>18.4<br>39	34.79<br>20.3<br>39	33.11<br>19.4<br>37	32.70<br>19.6<br>43	35.15<br>21.3<br>39	-
**ACT**											
Mean<br>ICBT<br>SD<br>N	15.13<br>4.3<br>46	16.70<br>4.0<br>46	17.31<br>4.2<br>45	17.64<br>4.3<br>44	18.28<br>4.3<br>43	19.41<br>3.8<br>44	18.62<br>4.8<br>42	18.70<br>4.4<br>44	19.13<br>3.2<br>45	19.95<br>4.2<br>43	20.20<br>3.6<br>40
Mean<br>TAU+ME<br>SD<br>N	16.39<br>4.0<br>44	18.35<br>4.1<br>43	18.22<br>4.5<br>41	18.18<br>4.8<br>39	18.40<br>4.8<br>40	18.85<br>5.1<br>39	18.13<br>5.3<br>39	18.49<br>5.4<br>37	17.91<br>4.7<br>43	17.56<br>4.4<br>39	-
**FAS**											
Mean<br>ICBT<br>SD<br>N	29.46<br>12.1<br>46	29.28<br>12.1<br>46	27.76<br>12.6<br>45	24.64<br>10.8<br>44	23.05<br>12.3<br>43	21.09<br>13.1<br>44	20.74<br>13.8<br>42	17.93<br>12.9<br>44	16.04<br>11.6<br>45	12.53<br>12.0<br>43	13.88<br>12.1<br>40
Mean<br>TAU+ME<br>SD<br>N	23.93<br>11.8<br>44	23.19<br>10.8<br>43	21.98<br>11.8<br>41	22.23<br>13.5<br>39	21.73<br>12.1<br>40	20.92<br>12.6<br>39	22.44<br>13.0<br>39	20.59<br>13.1<br>37	20.74<br>13.5<br>42	20.62<br>12.6<br>39	-
**ABC**											
Mean<br>ICBT<br>SD<br>N	27.33<br>12.0<br>46	27.24<br>10.6<br>46	26.44<br>10.5<br>45	25.00<br>10.0<br>44	23.56<br>10.2<br>43	23.09<br>10.9<br>44	23.48<br>11.1<br>42	21.07<br>9.7<br>44	19.47<br>9.4<br>45	17.51<br>8.8<br>43	18.45<br>9.0<br>40
Mean<br>TAU+ME<br>SD<br>N	26.82<br>11.1<br>44	28.02<br>12.0<br>43	26.39<br>12.6<br>41	27.18<br>13.7<br>39	26.27<br>12.3<br>40	26.46<br>13.3<br>39	26.44<br>12.9<br>39	26.24<br>13.9<br>37	26.15<br>13.0<br>41	25.79<br>12.7<br>39	-
**PSS10**											
Mean<br>ICBT<br>SD<br>N	18.20<br>7.1<br>46	17.11<br>6.6<br>46	17.16<br>7.0<br>45	15.75<br>7.8<br>44	15.84<br>7.3<br>43	14.25<br>8.2<br>44	16.48<br>8.9<br>42	15.84<br>8.4<br>44	13.69<br>8.3<br>45	13.43<br>8.4<br>42	13.38<br>8.1<br>40
Mean<br>TAU+ME<br>SD<br>N	18.14<br>7.1<br>44	18.09<br>5.5<br>43	16.98<br>7.5<br>41	16.74<br>8.1<br>39	16.32<br>7.4<br>40	15.69<br>6.8<br>39	15.49<br>7.2<br>39	15.19<br>7.5<br>37	16.54<br>7.3<br>41	15.87<br>7.4<br>38	-

Note. CAS=Catastrophizing about Asthma Scale; ACT=Asthma Control Test; FAS=Fear of Asthma Symptoms Scale; ABC=Asthma Behavior Checklist; PSS10=Perceived Stress Scale; *Increase in ACT and BBQ shows improvement.

Table S6. Summary of self-reported outcome means for outcomes *not* assessed weekly.

Mean<br>SD<br>N	Baseline	W 8	W 16	W 26
**BBQ**				
ICBT Mean<br>SD<br>N	54.43<br>20.0<br>46	62.82<br>19.1<br>45	66.65<br>19.6<br>43	65.05<br>20.0<br>40
TAU+ME Mean<br>SD<br>N	55.41<br>19.8<br>44	57.60<br>20.6<br>40	56.54<br>19.8<br>39	-
**ASI**				
ICBT Mean<br>SD<br>N	22.39<br>10.8<br>46	16.16<br>10.5<br>45	13.26<br>9.2<br>43	13.20<br>10.1<br>40
TAU+ME Mean<br>SD<br>N	22.61<br>11.7<br>44	21.95<br>12.3<br>41	19.23<br>11.2<br>39	-
**PSWQ**				
ICBT Mean<br>SD<br>N	52.91<br>13.1<br>46	49.60<br>13.5<br>45	46.71<br>11.7<br>42	46.27<br>13.3<br>40
TAU+ME Mean<br>SD<br>N	52.89<br>12.6<br>44	54.58<br>12.0<br>40	52.45<br>13.1<br>38	-
**SHAI**				
ICBT Mean<br>SD<br>N	21.52<br>8.5<br>46	17.82<br>8.7<br>45	15.70<br>8.1<br>43	17.12<br>9.2<br>40
TAU+ME Mean<br>SD<br>N	20.14<br>9.8<br>44	18.68<br>9.7<br>40	18.44<br>10.0<br>39	-
**ISI**				
ICBT Mean<br>SD<br>N	11.37<br>6.8<br>46	9.89<br>6.4<br>45	8.76<br>6.4<br>42	9.34<br>6.4<br>38
TAU+ME Mean<br>SD<br>N	10.61<br>6.2<br>44	10.55<br>6.8<br>40	9.92<br>5.6<br>38	-
**PHQ-9**				
ICBT Mean<br>SD<br>N	6.72<br>4.6<br>46	6.07<br>4.4<br>45	4.93<br>5.4<br>42	5.83<br>4.3<br>40
TAU+ME Mean<br>SD<br>N	6.93<br>4.8<br>44	6.90<br>5.0<br>40	6.68<br>5.4<br>38	-

Note. ASI=Anxiety Sensitivity Indes; PSWQ=Penn State Worry Questionnaire; BBQ=Brunnsviken Brief Quality of Life; HAIS=Health Anxiety Index-short scale; PHQ9=Patient Health Questionnaire; ISI=Insomnia Severity Index;

Table S7. Standardised effect sizes for each outcome.

Effect-sizes were estimated using linear regression with change from baseline as the outcome, controlling for treatment and baseline score. Weekly slope differences were estimated using mixed-effects models with standardised scores as the outcome and covariates for treatment, time, and their interaction. The slope reflects weekly change, and effect sizes represent the difference between the two arms in estimated weekly change, in standardised units, over the 16-week period.

Outcome	SMD 0-16 weeks β (95% CI)	Weekly SMD β (95% CI)
**CAS**	-1.17 (-1.61, -0.73)	-0.09 (-0.10, -0.07)
**ACT***	0.67 (0.24, 1.10)	0.05 (0.03, 0.07)
**FAS**	-0.90 (-1.28, -0.53)	-0.07 (-0.09, -0.06)
**ABC**	-0.74 (-1.07, -0.40)	-0.05 (-0.06, -0.04)
**BBQ***	0.57 (0.21, 0.94)	0.03 (0.01, 0.05)
**ASI**	-0.56 (-0.81, -0.31)	-0.03 (-0.05, -0.02)
**PSWQ**	-0.44 (-0.70, -0.18)	-0.02 (-0.04, -0.01)
**SHAI**	-0.48 (-0.72, -0.24)	-0.03 (-0.04, -0.01)
**ISI**	-0.33 (-0.63, -0.02)	-0.02 (-0.04, 0.00)
**PSS10**	-0.34 (-0.74, 0.06)	-0.02 (-0.04, 0.00)
**PHQ9**	-0.37 (-0.86, 0.11)	-0.02 (-0.05, 0.01)

Note. SMD=Standardized mean difference. CAS=Catastrophizing about Asthma Scale; ACT=Asthma Control Test; FASS=Fear of Asthma Symptoms Scale; ABC8=Asthma Behavior Checklist; PSS10=Perceived Stress Scale; ASI=Anxiety Sensitivity Indes; PSWQ=Penn State Worry Questionnaire; BBQ=Brunnsviken Brief Quality of Life; HAIS=Health Anxiety Index-short scale; PHQ9=Patient Health Questionnaire; ISI=Insomnia Severity Index; * Increase in ACT and BBQ indicates improvement in symptoms. For all other outcomes a decrease indicates improvement. Participants included in models: For change from baseline, N_{ICBT} = 43 and N_{TAU+ME} = 38 except for PSWQ and ISI, PSS10 & PHQ9 (42, 38). For mixed effects model, N_{ICBT} = 46 and N_{TAU+ME} = 43

2.3 Treatment completion and dose-response

Table S8. Summary of CAS scores by number of modules completed

**Number of sessions**	**N**	**CAS at baseline** Mean (SD)	**CAS at week 16** Mean (SD)	**Change from baseline to week 16** Mean (SD)
0	44	37.4 (15.3)	35.2 (21.3)	-2.5 (14.9)
1	4	55.2 (20.2)	20.3 (16.5)	-35.3 (23.7)
2	0	-	-	-
3	2	28.5 (17.7)	29.0 (14.1)	0.5 (3.5)
4	4	35.8 (20.2)	16.5 (14.4)	-19.2 (24.6)
5	3	39.0 (10.8)	10.5 (13.4)	-27.0 (1.4)
6	3	40.3 (9.7)	31.7 (27.4)	-8.7 (21.4)
7	5	44.4 (17.9)	24.2 (12.3)	-20.2 (17.9)
8	25	45.9 (14.8)	19.5 (17.5)	-26.0 (15.2)

Note. CAS=Catastrophizing about Asthma Scale. All randomised to ICBT arm completed at least one module. Hence, all individuals who completed no modules were in the TAU+ME arm.

Among participants who completed fewer than four modules (n = 6), reported reasons for discontinuation included lack of time, reduced motivation, perceived improvement, limited fit with the treatment content, illness, or other life circumstances. To protect participant privacy, individual reasons are not presented in detail.

Table S9. Regression results of CAS scores by number of sessions attended

**Parameter**	**Estimate**	**95% CI**	**P-value**
Time (weeks)	-0.13	(-0.29, 0.04)	0.145
Treatment	0.61	(-0.33, 1.56)	0.205
Time x treatment interaction	-0.18	(-0.22, -0.15)	< 0.001
Constant	34.31	(29.70, 38.92)	< 0.001
**Random effects parameters**			
Participant-level variance		252.20	
Residual variance		72.78	
ICC		0.78	

Note. Results from a mixed effects linear regression model using CAS data from all timepoints up to week 16 as the outcome. Model includes an interaction between number of sessions attended (as a continuous variable) and weeks since start (also a continuous variable). No other variables were included in the model. A random intercept for each participant is included.

2.4 Subjective adequate relief

Participants in both groups rated overall symptom changes from baseline to 8 weeks (posttreatment) using a 7-point Likert scale, ranging from 0 ("much worse") to 6 ("much improved").

Figure S1. Subjective symtom relief at week 8 in both group internet-delivered cognitive behaviour therapy (ICBT and treatment as usual (TAU; which also included the same medical education as in ICBT).

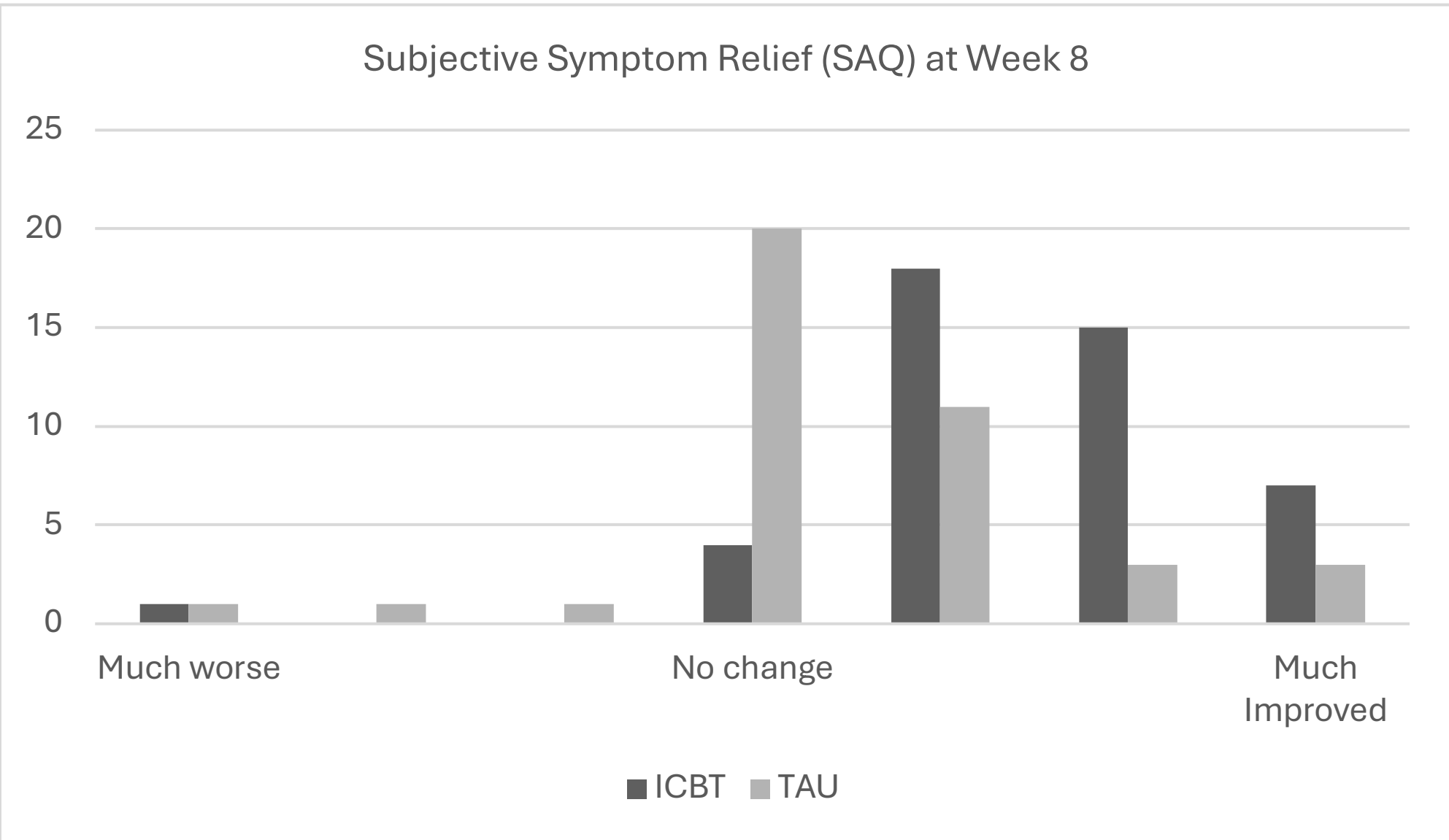

2.5 Adverse events

At week eight (after the treatment period), participants who received ICBT were asked about adverse events during the treatment, specifically, *"Anything that negatively impacted or was difficult during the treatment."* Nine participants reported treatment-or study-related difficulties.

- Two participants felt stressed by the treatment pace and number of questions.
- One found the study questions irrelevant to her situation.
- Three reported discomforts from increased awareness of asthma and anxiety.
- Two experienced somatic symptoms (dyspnoea, palpitations, and disturbed sleep).

- Additionally, one participant noted that catching a cold increased anxiety and worsened symptoms, until she started to use the situation as an exposure exercise.

Most reported effects were mild and temporary. However, the participant with dyspnoea found it highly distressing at the time but without any lasting effect, whereas the participant with palpitations and sleep disturbances reported moderate effects with some lingering impact. Both participants discontinued treatment early but continued to complete the assessments.

2.6 Change in Asthma Medication

At weeks 8 and 16, participants were asked whether they had changed their medication in the past 8 weeks.

Table S10. Change in asthma medication from pretreatment to 16 weeks

**Medication change**	**ICBT (N=45) 0-8 weeks**	**TAU+ ME (N=38) 0-8 weeks**	**ICBT (N=43) 8-16 weeks**	**TAU+ME (N=37) 8-16 weeks**
**No change**, n (%)	27 (62%)	25 (66%)	35 (81%)	27 (73%)

Note. *Most changes were made in consultation with a physician or asthma nurse. ICBT=Internet-delivered cognitive behaviour therapy; TAU+ME=treatment as usual + medical education about asthma*

Supplement to

Online cognitive behaviour therapy for asthma-related anxiety: a randomised controlled trial

References

1 Sheehan DV, Lecrubier Y, Sheehan KH, Amorim P, Janavs J, Weiller E *et al.* The Mini-International Neuropsychiatric Interview (M.I.N.I.): the development and validation of a structured diagnostic psychiatric interview for DSM-IV and ICD-10. *J Clin Psychiatry* 1998; **59 Suppl 20**: 22-33-quiz 34–57.

2 Ljungberg H, Carleborg A, Gerber H, Öfverström C, Wolodarski J, Menshi F *et al.* Clinical effect on uncontrolled asthma using a novel digital automated self-management solution: a physician-blinded randomised controlled crossover trial. *Eur Respir J* 2019; **54**: 900983.

3 Myers L, Bellander M, Ljungberg H, Isachsen M, Eduards M, Lindman M *et al.* Late Breaking Abstract - Assessing the diagnostic accuracy of home spirometry system for asthma diagnosis. *M-Heal.e-Heal* 2024; : PA5199.

4 Bonnert M, Andersson E, Serlachius E, Manninen I-K, Bergström SE, Almqvist C. Exposure-based cognitive behavior therapy for anxiety related to asthma: A feasibility study with multivariate baseline design. *Scand J Psychol* 2020; **61**: 827–834.

5 Bonnert M, Särnholm J, Andersson E, Bergström SE, Lalouni M, Lundholm C *et al.* Targeting excessive avoidance behavior to reduce anxiety related to asthma: A feasibility study of an exposure-based treatment delivered online. *Internet Interventions* 2021; **25**: 100415.
